# Contrasting pandemic era trajectories of dengue; chikungunya; and hand, foot, and mouth disease in Thailand using national surveillance findings, 2016–2025

**DOI:** 10.1016/j.ijregi.2026.101037

**Published:** 2026-10-07

**Authors:** Wannarat Pongpirul, Mohamed Mustaf Ahmed, Krit Pongpirul

**Affiliations:** 1 Department of Disease Control, Bamrasnaradura Infectious Diseases Institute, Ministry of Public Health, Nonthaburi, Thailand; 2 Faculty of Medicine and Health Sciences, SIMAD University, Mogadishu, Somalia; 3 Faculty of Medicine, School of Global Health, Chulalongkorn University, Bangkok, Thailand; 4 Faculty of Medicine, Center of Excellence in Preventive and Integrative Medicine and Department of Preventive and Social Medicine, Chulalongkorn University, Bangkok, Thailand; 5 Clinical Research Center, Bumrungrad International Hospital, Bangkok, Thailand; 6 Department of Infection Biology and Microbiomes, Faculty of Health and Life Sciences, University of Liverpool, Liverpool, United Kingdom

**Keywords:** Dengue, Chikungunya, Hand, foot, and mouth disease, COVID-19, Interrupted time series design

## Abstract

**Objectives:**

To compare the pandemic era trajectories of dengue; chikungunya; and hand, foot, and mouth disease (HFMD) in Thailand

**Methods:**

We conducted a national ecological interrupted time series analysis using weekly surveillance data from all 77 Thai provinces from 2016 to 2025. Segmented quasi-Poisson regression with Fourier terms for annual seasonality estimated the level and slope changes at a calendar-based 2020 break point; sensitivity analyses used later break points.

**Results:**

The analysis included 40579 province-week observations including 790263 dengue, 32265 chikungunya, and 713822 HFMD cases. The estimated level incidence rate ratios at week 1 of the 2020 break point were 0.39 for dengue (95% confidence interval [CI] 0.32-0.48), 0.54 for chikungunya (95% CI 0.41-0.71), and 0.51 for HFMD (95% CI 0.38-0.69). Dengue rebounded above pre-pandemic levels by 2023, chikungunya declined after the 2018-2020 outbreak, and HFMD’s mean annual incidence in 2023–2025 exceeded the pre-pandemic baseline by approximately 26%.

**Conclusions:**

These contrasting trajectories support the need for comparative multi-disease surveillance. Ecological designs cannot separate the contribution of transmission ecology from epidemic history or other time-varying factors.

## Highlights

• A total of 10 years of nationwide surveillance data from all 77 Thai provinces were analyzed.
• All three diseases showed level reductions at the calendar-based 2020 break point.
• Dengue rebounded above baseline; chikungunya declined after its epidemic wave.
• Hand, foot, and mouth disease recovered rapidly and exceeded its pre-pandemic incidence.
• Observed differences cannot be attributed independently to transmission ecology.

## Introduction

The COVID-19 pandemic changed population movement, social contact, and access to routine health services worldwide. These changes affected the transmission and detection of infectious diseases, with effects that varied across pathogens [[1], [2], [3]]. Dengue and chikungunya differ from hand, foot, and mouth disease (HFMD) in their transmission routes and affected populations; therefore, their trajectories during this shared disruption may differ.

Dengue, chikungunya, and HFMD are major notifiable infections in Thailand that can be compared across broad transmission categories. Dengue and chikungunya are transmitted primarily by *Aedes* mosquitoes and remain important public health concerns in tropical and subtropical regions. Thailand is hyperendemic for dengue and experienced a major nationwide chikungunya outbreak during 2018–2019, driven by the East/Central/South African Indian Ocean lineage [[4], [5], [6], [7]]. HFMD predominantly affects young children and is caused by multiple enteroviruses, including enterovirus A71 and coxsackieviruses. Transmission occurs via fecal–oral spread and contact with respiratory secretions, vesicular fluid, or contaminated surfaces [8,9].

Differences in epidemic history and affected populations complicate comparisons of pandemic era trajectories. For example, population aging has been implicated in the changing age distribution of dengue cases in Thailand [10]. Using the same surveillance system and observation period provides a common setting for comparison but cannot by itself separate transmission ecology from other disease-specific influences.

Interrupted time series analysis provides a useful framework for estimating level and slope changes around a specified interruption [11]. Using 10 years of weekly surveillance data from all 77 provinces of Thailand, we characterized the decadal trajectories of dengue, chikungunya, and HFMD; quantified level and slope changes around specified pandemic-era break points; and compared trajectories across broad transmission categories during a common period of population-level disruption.

## Methods

### Study design and setting

We conducted a national ecological interrupted time series analysis using weekly notifiable disease surveillance data from all 77 provinces of Thailand during 2016–2025. The COVID-19 pandemic was treated as a population-level disruption affecting diseases in broad transmission categories within a shared surveillance system. This study followed the Strengthening the Reporting of Observational Studies in Epidemiology (STROBE) guidelines. The completed STROBE checklist is included as Supplementary Table S1 in Supplementary File S1.

### Data sources

Weekly province-level notifications from January 1, 2016 to December 31, 2025 were obtained from Thailand’s Ministry of Public Health 506 notifiable disease surveillance system, which receives mandatory reports from public and private health care facilities nationwide. The records used the International Classification of Diseases, Tenth Revision codes for dengue fever (A90), dengue hemorrhagic fever (A91), dengue shock syndrome (A91.1), chikungunya (A92.0), and HFMD (B08.4 and B08.5).

Year-specific provincial population denominators were obtained from the Department of Provincial Administration. Province boundary files used for spatial analyses were obtained from the Royal Thai Survey Department through the United Nations Office for the Coordination of Humanitarian Affairs administrative boundary data set.

The harmonized province-week records constitute the Thailand release of the Temporal Recent Epidemiology of Notifiable Diseases in Southeast Asia and are publicly available in Zenodo [12]. The data set contained 40579 province-week observations that spanned 527 epidemiologic weeks. No province-week records were missing, and no imputation was required; however, this does not establish complete ascertainment of infections. A preprint version of this manuscript has been posted on medRxiv [13].

### Outcome definitions

The primary outcome was the weekly incidence per 100000 population, calculated using weekly case counts and year-specific provincial population denominators. Dengue analyses used a composite series combining dengue fever, dengue hemorrhagic fever, and dengue shock syndrome. Chikungunya and HFMD were analyzed separately. For descriptive comparisons, diseases were grouped into broad transmission categories, comprising vector-borne (dengue and chikungunya) and predominantly transmitted between people (HFMD). This grouping does not isolate the transmission mechanism from differences in age distribution, causative viruses, immunity, or epidemic history.

Three periods were defined for descriptive analyses: pre-pandemic (2016–2019), pandemic (2020–2022), and post-pandemic (2023-2025). The 2023 boundary retained the entire calendar year after the designated 2020-2022 phase. “Post-pandemic” is an operational label and does not imply that the circulation of the virus causing COVID-19 or its effects ended in 2023. The primary interrupted time series break point was January 1, 2020 (reporting week 1), aligning the model boundary with the descriptive periods rather than the introduction of restrictions. Alternative model thresholds were March 22, 2020 and October 12, 2020. The first observations included after these thresholds were March 22, 2020 (reporting week 13) and October 18 (reporting week 43), respectively. The January break point places weeks preceding restrictions in the post-interruption segment, limiting the attribution of its level change to COVID-19–related societal disruption.

### Statistical analysis

We summarized the temporal patterns using weekly incidence trajectories, seasonal distributions, province-level incidence rates, and phase-specific incidence estimates. We used segmented quasi-Poisson regression with Fourier terms for annual seasonality and population offsets for changes in population size. Each model estimated an immediate level change and a change in slope at the specified break point. We reported the incidence rate ratio (IRR) for each level change and the annualized percentage for each slope change, with a 95% confidence interval (CI) for each estimate. Slope changes were relative to the pre-interruption slope, not the absolute annual post-interruption trends. The model specification is provided in the Supplementary Methods.

Seasonal reference trajectories were generated from pre-pandemic data to visualize the deviations from the baseline disease activity. For chikungunya, the reference trajectory used the low-incidence 2016–2017 period, whereas the segmented model used the full series, including the 2018–2019 outbreak in its pre-interruption trends. This descriptive reference trajectory does not isolate the pandemic effect from the epidemic dynamics. Exploratory cross-correlations of detrended log-transformed rates were used to describe the temporal associations between diseases. To facilitate comparisons across broad transmission categories, 8-week smoothed weekly incidence rates were indexed to the pre-pandemic mean of the smoothed series for each category.

### Sensitivity analyses

Sensitivity analyses re-estimated the same disease-specific models at the two alternative date thresholds. The sensitivity analyses are based on the March 22, 2020 break point because it more closely approximates the timing of restriction implementation than the January break point. These analyses assess sensitivity to timing but do not control for epidemic exhaustion or other time-varying factors. All original analyses were performed using Python version 3.13.

### Ethics statement

This study used aggregated surveillance data and was determined to be exempt from ethical review by the institutional review board of Bamrasnaradura Infectious Diseases Institute, Thailand (Project No. S002 h/69_Exempt; determination dated February 27, 2026). The data set contained no individual-level identifiers.

## Results

### Surveillance records and disease groupings

The analysis included 40579 province-week observations across 77 Thai provinces and 527 epidemiological weeks during 2016-2025, comprising 790263 dengue, 32265 chikungunya, and 713822 HFMD cases. Dengue and chikungunya formed the vector-borne category and HFMD, a pediatric enteroviral disease, represented infections that were predominantly transmitted between people.

The annual case counts varied across all three diseases. Dengue cases declined to 10524 in 2021 before increasing to 160841 in 2023. Chikungunya had 11721 cases in 2019, with substantial activity continuing into 2020 before it declined. The number of HFMD cases fell to 19007 in 2021 before rebounding to 115264 in 2025 (Supplementary Table S2).

The national population denominator was relatively stable at 65.9 million in 2016, 66.6 million in 2019, and 65.8 million in 2025. Therefore, changes in crude incidence were mainly driven by reported case counts rather than by changes in total population size.

### National temporal trajectories

The 8-week centered rolling mean of the national weekly incidence per 100000 population showed different peak timings for each disease. Dengue cases peaked in August 2023, after a marked decline during the pandemic. Chikungunya cases peaked in July 2020 as the 2018-2019 outbreak continued into 2020. HFMD peaked in August 2022, after a decline in 2020-2021 (Figure 1).

**Figure 1:**
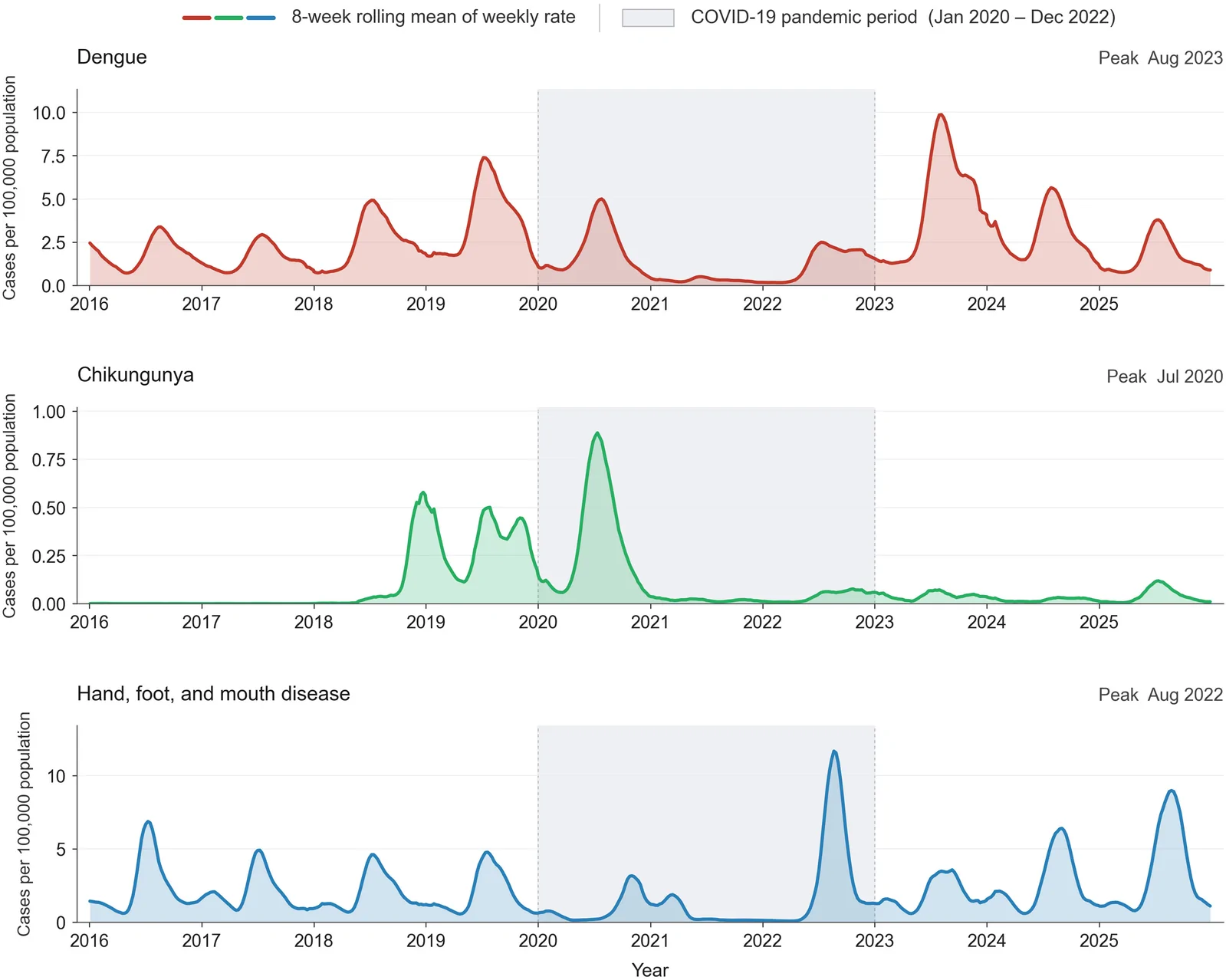
National weekly incidence per 100000 population for dengue; chikungunya; and hand, foot, and mouth disease, Thailand, 2016–2025. Lines show 8-week centered rolling means. Shading denotes the operational pandemic study period (2020–2022).

The composition of dengue severity shifted progressively over the decade. Dengue fever increased from approximately 60% of notifications in 2016 to 70-75% during the pandemic and post-pandemic periods, whereas dengue hemorrhagic fever declined from approximately 39% to 24-29%. Dengue shock syndrome remained stable at approximately 1% throughout the study period (Supplementary Figure S1).

The mean annual dengue incidence fell from 126.4 per 100000 population before the pandemic to 65.4 during 2020–2022 (−48.3%), then rose to 164.2 during 2023–2025 (+29.8%). Chikungunya incidence increased from 5.8 to 6.7 per 100000 (+16.3%) before declining to 1.8 (−68.1% decline). HFMD incidence decreased from 109.0 to 77.1 per 100000 (−29.3%) and subsequently increased to 137.5 (+26.1%). All percentage changes were relative to those in 2016–2019 (Supplementary Table S3).

Dengue exhibited a consistent peak in July across all phases. HFMD peaked in July before the pandemic and in August after the pandemic. Chikungunya exhibited a temporal shift, with peak incidence occurring in November before the pandemic and July during and after the pandemic, with the pandemic period peak reflecting the activity in 2020 (Figure 2).

**Figure 2:**
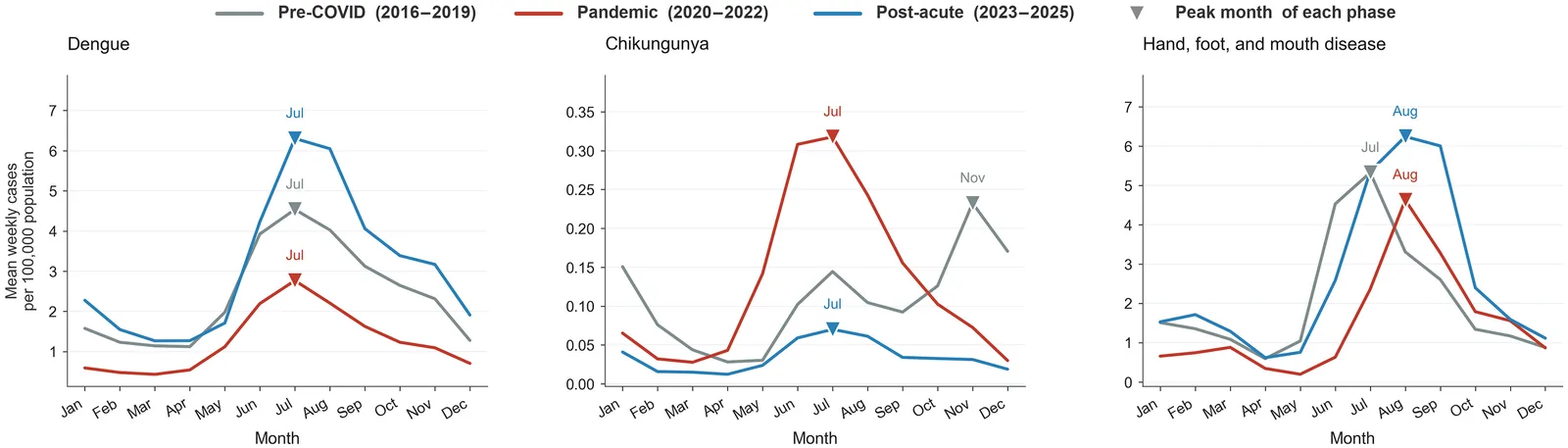
Seasonal profiles of mean weekly incidence per 100000 population by calendar month and study period. Within each year and month, cases were divided by the sum of weekly population denominators; these mean weekly rates were then averaged across years in each period. “Post-pandemic” denotes the operational 2023–2025 study period and does not imply an end to COVID-19 effects.

### Geographic distribution

The mean annual incidence varied geographically. Dengue incidence was high in northern provinces, including Mae Hong Son, Chiang Rai, and Chiang Mai, and along the eastern coast in Rayong and Trat. Chikungunya incidence was high in Chanthaburi and in southern provinces, including Songkhla and Pattani. HFMD incidence was high in the northern and eastern coastal provinces, partly overlapping with dengue, and was lower in the deep south (Figure 3a).

**Figure 3:**
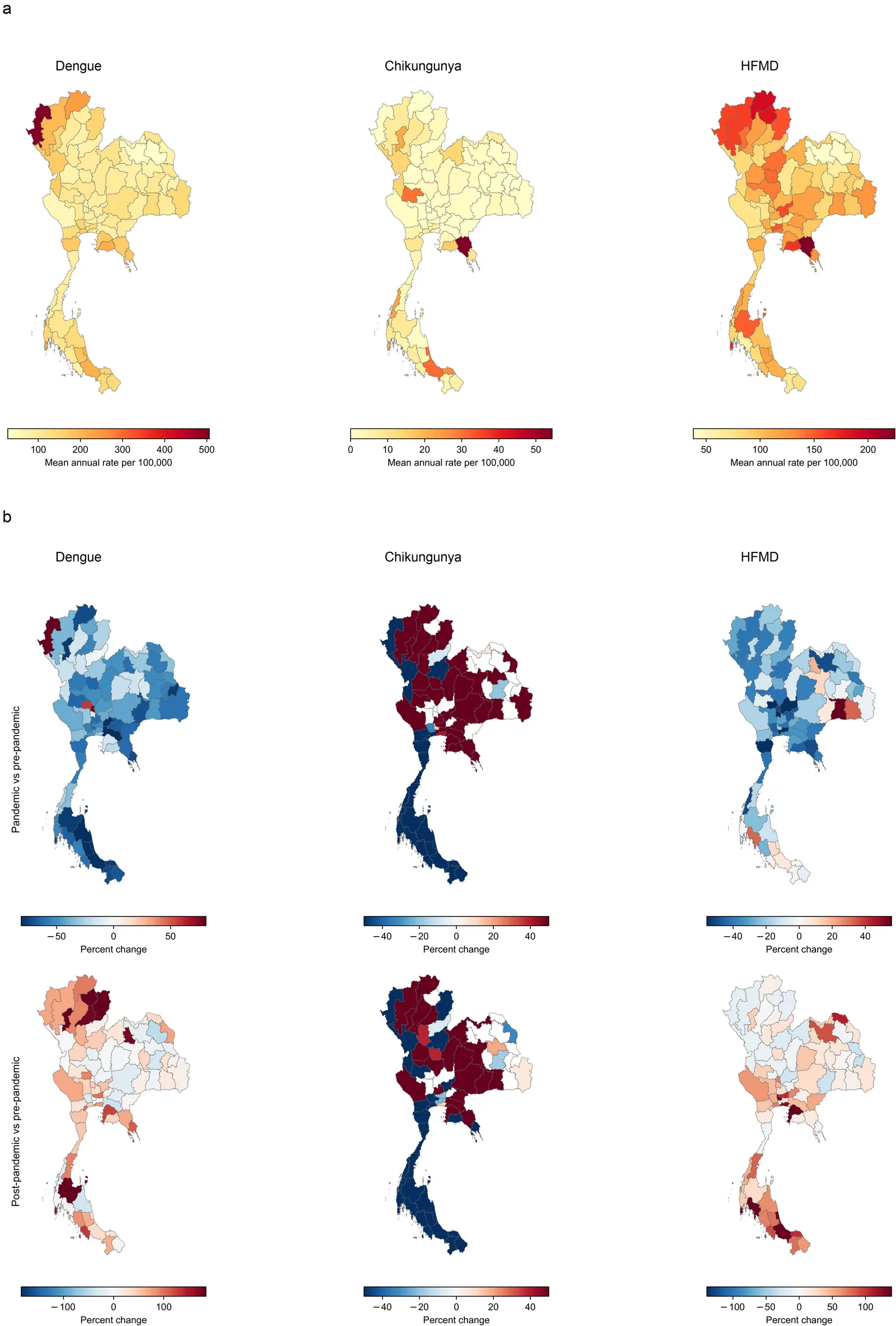
(a) Province-level mean annual incidence per 100000 population for dengue, chikungunya, and HFMD, Thailand, 2016–2025. The maps show average annual burden over the study period, not 10-year cumulative incidence. (b) Province-level percentage change in mean annual incidence during 2020–2022 and 2023–2025 relative to 2016–2019. “Post-pandemic” denotes the operational 2023–2025 study period. Province boundaries were provided by the Royal Thai Survey Department and distributed through the United Nations Office for the Coordination of Humanitarian Affairs. Abbreviation: HFMD, hand, foot, and mouth disease.

The highest-burden provinces varied by disease. The mean annual dengue incidence was highest in Mae Hong Son (504.1 per 100000 population), Chiang Rai (239.1), and Rayong (214.7); chikungunya incidence was highest in Chanthaburi (54.4), Songkhla (30.7), and Uthai Thani (30.2); and HFMD incidence was highest in Chanthaburi (224.9), Chiang Rai (189.6), and Phayao (182.7). Chanthaburi ranked among the highest-incidence provinces for chikungunya and HFMD (Supplementary Table S4).

Compared with the pre-pandemic period, most provinces experienced a decline in dengue and HFMD incidence during the pandemic. Post-pandemic incidence increased across most of northern and central Thailand for dengue and HFMD, whereas chikungunya incidence remained below pre-pandemic levels in most provinces (Figure 3b).

### Interrupted time series analyses

Across 2020–2025, the observed case totals were below their seasonal reference totals by 9.2% for dengue and 1.5% for HFMD. The fitted HFMD trajectory exceeded its seasonal reference during 2024–2025. Chikungunya remained substantially above its low-incidence 2016–2017 reference, reflecting the persistence of the epidemic into 2020. These deviations describe departures from seasonal references and cannot be interpreted as attributable pandemic effects (Figure 4).

**Figure 4:**
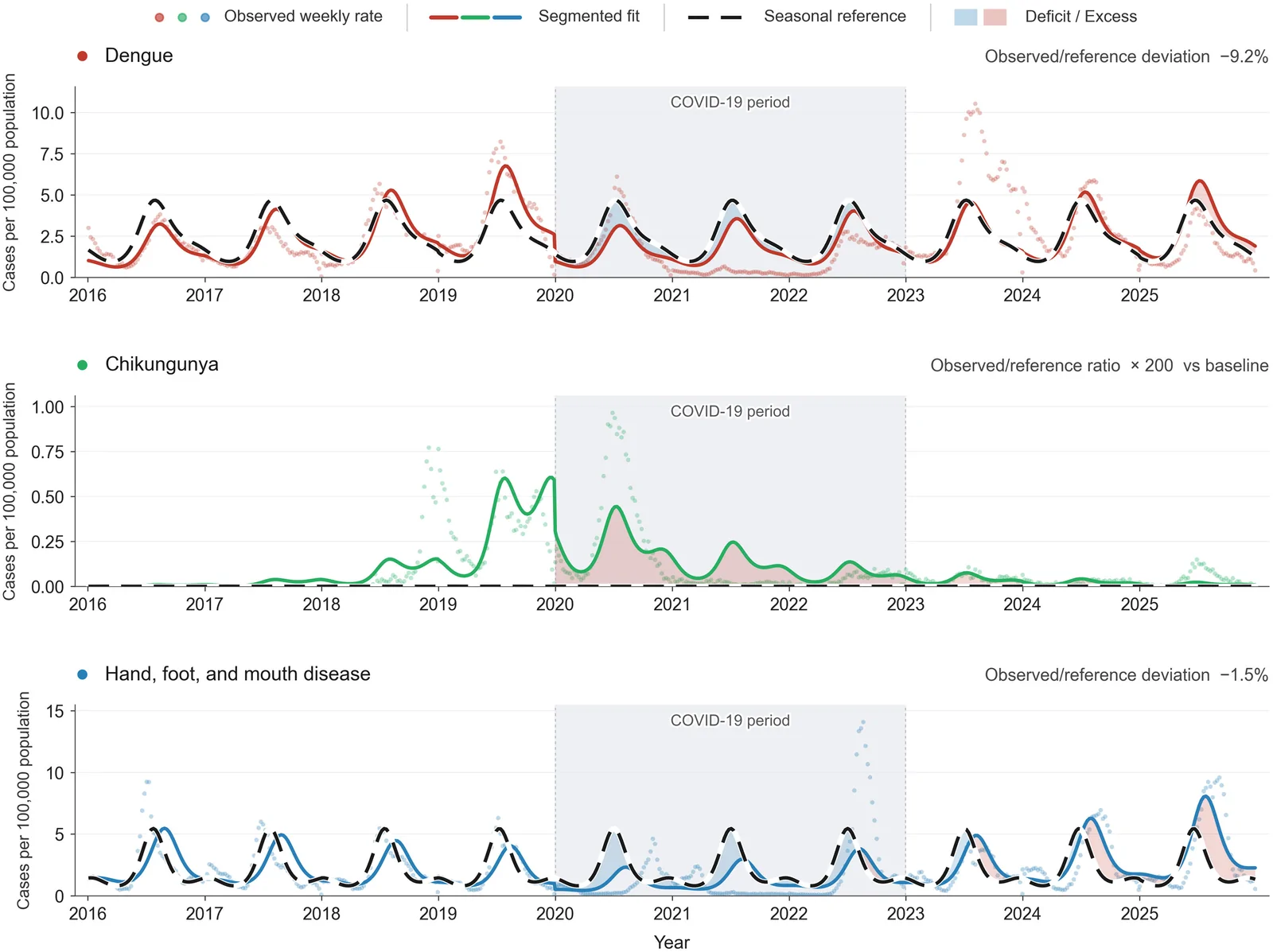
Segmented interrupted time series fits and seasonal reference trajectories, Thailand, 2016–2025. Dots indicate observed weekly rates, solid lines indicate segmented fits, dashed lines indicate seasonal references fitted to 2016–2019 for dengue and HFMD and to 2016–2017 for chikungunya. Shading marks 2020–2022. The displayed deviations compare observed and reference case totals across 2020–2025; the chikungunya multiplier is the observed/reference ratio. These comparisons do not identify attributable pandemic effects. Abbreviation: HFMD, hand, foot, and mouth disease.

At the January 1, 2020 break point, the estimated level IRRs were 0.39 for dengue (95% CI 0.32-0.48; *P* < 0.001), 0.54 for chikungunya (95% CI 0.41-0.71; *P* < 0.001), and 0.51 for HFMD (95% CI 0.38-0.69; *P* < 0.001). These estimates indicate reductions relative to the projected pre-interruption trends.

Annualized slope changes relative to the pre-interruption trends were −11.4% for dengue (95% CI −18.2% to −3.9%; *P* = 0.003), −85.9% for chikungunya (95% CI −88.6% to −82.6%; *P* < 0.001), and +41.9% for HFMD (95% CI +28.2% to +57.1%; *P* < 0.001) (Table 1). The chikungunya estimate describes a change relative to the pre-2020 slope, which includes the epidemic upswing rather than an 85.9% annual reduction in the observed incidence.

**Table 1:** Interrupted time-series estimates at January 1, 2020 calendar break point.

Disease	Effect	Coefficient (log scale)	Standard error	Magnitude (95% CI)	*P*-value
Dengue	Immediate level change	−0.945	0.106	IRR 0.39 (0.32-0.48)	<0.001
	Slope change relative to pre-interruption trend	−0.002	0.001	−11.4% per year (−18.2% to −3.9%)	0.003
Chikungunya	Immediate level change	−0.613	0.138	IRR 0.54 (0.41-0.71)	<0.001
	Slope change relative to pre-interruption trend	−0.038	0.002	−85.9% per year (−88.6% to −82.6%)	<0.001
Hand, foot, and mouth disease	Immediate level change	−0.673	0.153	IRR 0.51 (0.38-0.69)	<0.001
	Slope change relative to pre-interruption trend	0.007	0.001	41.9% per year (28.2-57.1%)	<0.001

Estimates are from segmented quasi-Poisson models with two annual Fourier harmonics and a log-population offset. The level change is the step at the specified calendar break point. Annualized slope change equals 100 × [exp (52 × the slope-change coefficient) − 1], relative to the pre-interruption slope; it is not the absolute post-interruption annual trend. The break point is not the date when restrictions began.

Abbreviations: CI, confidence interval; IRR, incidence rate ratio.

### Sensitivity to alternative interruption dates

The sensitivity analysis using a March 22, 2020 break point produced immediate IRRs of 0.39 (95% CI 0.32-0.49) for dengue, 0.47 (95% CI 0.35-0.62) for chikungunya, and 0.55 (95% CI 0.41-0.75) for HFMD (*P* < 0.001 for each). These estimates were similar to the January estimates of 0.39, 0.54, and 0.51. At the October 12, 2020 threshold, the IRRs were 0.37 for dengue and 0.05 for chikungunya (both *P* < 0.001), whereas the HFMD estimate was 1.30 (*P* = 0.077). Agreement was strongest between the January and March thresholds; the October threshold produced substantial changes for chikungunya and HFMD.

At the 22 March 2020 break point, the annualized slope changes relative to the pre-interruption trends were −8.5% for dengue (95% CI −15.4% to −0.9%), −84.2% for chikungunya (95% CI −87.1% to −80.6%), and +44.1% for HFMD (95% CI +30.4% to +59.2%). Chikungunya and HFMD retained the direction of slope change across thresholds, whereas the dengue estimate changed to +8.2% at the October threshold (Supplementary Table S5).

### Cross-disease temporal associations

Exploratory cross-correlations of detrended weekly log-transformed incidence were strongest at lag 0 for dengue and chikungunya (*r* = 0.44) and dengue and HFMD (*r* = 0.60). Chikungunya–HFMD correlations were weak across the examined lags, with the largest absolute correlation of −0.09 at lag −26 weeks, slightly outside the nominal reference band. The band does not account for serial dependence or testing across multiple lags; therefore, it should not be interpreted as a formal significance threshold (Figure 5a).

**Figure 5:**
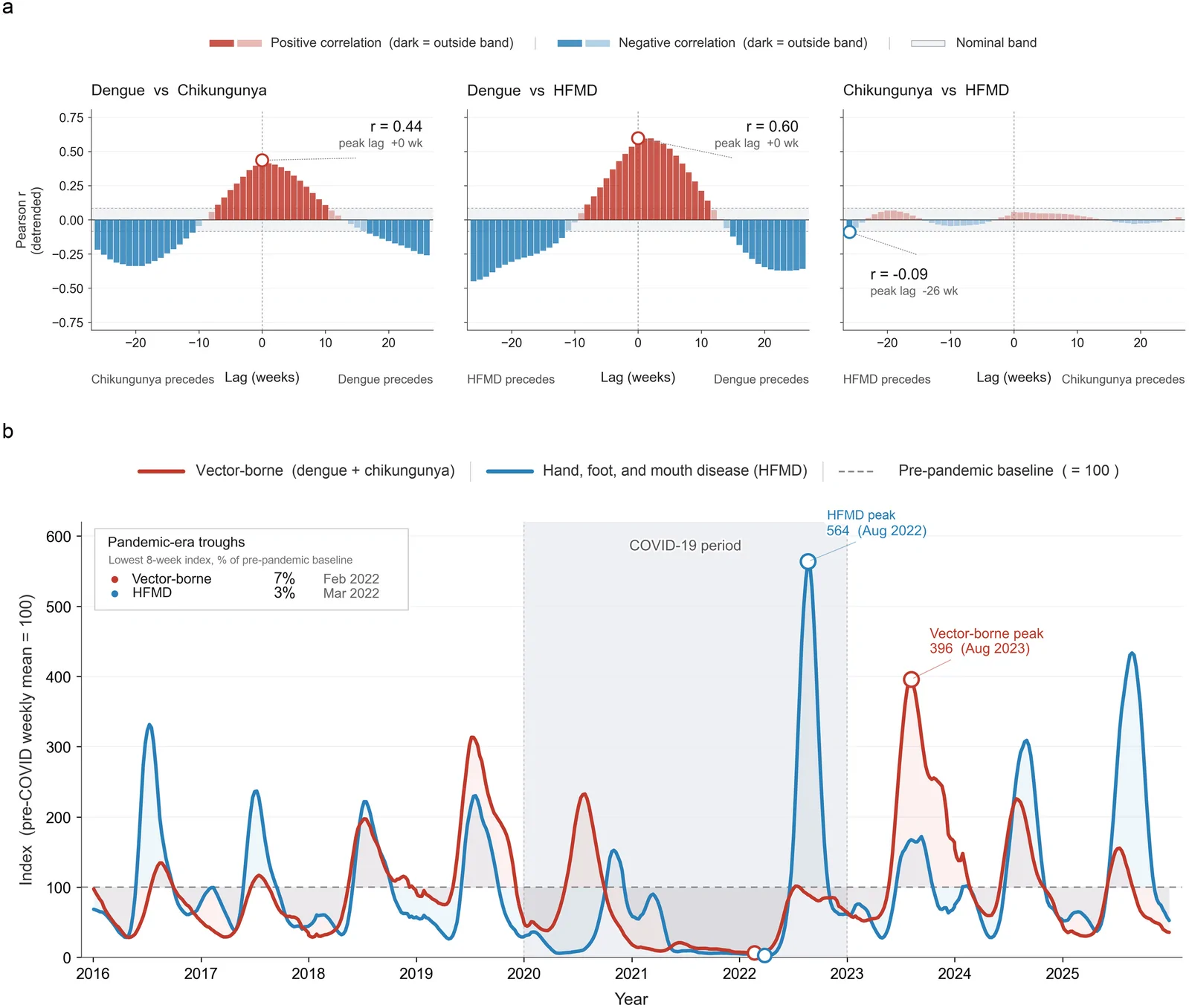
(a) Exploratory lag correlations of detrended national weekly log-transformed rates. Detrending subtracts a centered 52-week moving mean from log (1 + incidence); it does not remove seasonal cycles. The shaded nominal band is ±1.96/√527 (approximately ±0.085). It does not account for serial dependence or comparisons across multiple lags. Darker bars lie outside this descriptive band and should not be read as formal significance tests. (b) Indexed weekly trajectories across broad transmission categories comprising dengue plus chikungunya and HFMD. Each 8-week centered rolling mean is divided by its pre-pandemic mean and multiplied by 100. Shading denotes 2020–2022. This comparison does not isolate transmission mechanism from age, pathogen diversity, immunity, or epidemic history. Abbreviation: HFMD, hand, foot, and mouth disease.ss

When indexed to a pre-pandemic baseline of 100, the vector-borne category and HFMD declined sharply during 2020–2021 and subsequently exceeded the baseline levels. The HFMD rebound peaked in August 2022, approximately 1 year before the vector-borne peak in August 2023 (Figure 5b).

## Discussion

Dengue, chikungunya, and HFMD followed different pandemic era trajectories within Thailand’s surveillance system. Models estimated level reductions at week 1 of the 2020 breakpoint for all three diseases, with similar estimates at the March 22, 2020 break point. Dengue subsequently rebounded above pre-pandemic levels, chikungunya declined after the 2018–2020 epidemic, and HFMD recovered rapidly and exceeded baseline incidence. Because week 1 precedes restrictions, its estimated level changes cannot be interpreted as immediate effects of restrictions. The comparison describes differences across broad transmission categories without establishing transmission ecology as their cause.

The decline in dengue incidence and subsequent resurgence in Thailand occurred amid high global dengue activity. More than 10 million cases were reported across 176 countries by July 23, 2024 [14]. Our findings are consistent with the early pandemic decline in Thai dengue notifications [15]. However, in Singapore, notifications increased during 2020 despite movement restrictions [16], indicating that pandemic era dengue patterns varied with local conditions.

The chikungunya pattern requires caution. The major 2018–2019 outbreak and the role of immune responses in infection provide context for possible epidemic exhaustion [17,18]. In this data set, 11721 cases were reported in 2019 and 11334 in 2020, and a smoothed weekly peak occurred in July 2020. Therefore, the epidemic overlapped with the interruption rather than ending before it. The −85.9% estimate represents a slope change relative to the outbreak-influenced pre-2020 trend. This may substantially reflect epidemic exhaustion and regression toward the baseline, independent of COVID-19. These data cannot quantify the separate contributions of natural epidemic dynamics and pandemic-related changes, and shifting the breakpoint to March 22, 2020 does not resolve this confounding factor.

HFMD rebounded the earliest, with a smoothed peak in August 2022. Its decline during 2020–2021 and subsequent resurgence above the baseline are consistent with observations from China and the proposed immunity debt hypothesis for pediatric infections [[19], [20], [21]]. Changes in school and daycare attendance, population mixing, and susceptibility may have contributed; however, these factors were not measured. Changes in circulating enteroviruses or case ascertainment could also affect the observed trajectory.

Transmission ecology is one possible explanation for the differing trajectories. Human movement between households can contribute to dengue transmission [22]. However, this comparison did not measure the relevant exposures for each disease. HFMD also differs from dengue and chikungunya in terms of age distribution, pathogen diversity, and population immunity. The earlier recovery of HFMD is, therefore, compatible with several explanations and cannot establish an independent effect of transmission mode.

The study covered all 77 provinces over 10 years, with a complete province-week reporting grid and year-specific civil registration denominators for incidence calculations. The public data set also allows the analyses to be reproduced and extended.

The Ministry of Public Health 506 system uses passive surveillance and is subject to variation in health care–seeking behavior, diagnostic practices, and reporting completeness. Pandemic-related disruptions may have affected transmission and surveillance sensitivity. The aggregated data did not include serotype, genotype, or individual-level clinical information. Models were adjusted for time, seasonality, and population size but not for mobility, school closures, health care–seeking behavior, vector control, or population immunity, which may have changed differently across diseases. Without suitable controls or these covariates, an ecological interrupted time series cannot isolate the contribution of transmission ecology from concurrent changes [23]. The week 1 break point misclassified the onset of restrictions, with an uncertain direction of bias. For chikungunya, the preceding epidemic further prevented the attribution of the slope change to COVID-19. Residual serial correlation was not explicitly modeled; therefore, CIs may understate uncertainty. Changes in dengue severity classification may affect stratum-specific patterns, although combining all three reported strata reduces sensitivity to shifts between categories.

These patterns support the maintenance of multi-disease surveillance in Thailand and similar Southeast Asian settings. Pandemic period declines do not imply sustained reductions in the reported disease burden. Dengue resurgence reinforces the need for continued vector control and appropriate vaccine use [24]. The HFMD rebound also supports continued enterovirus surveillance and the consideration of preventive strategies for young children [25].

## Conclusion

Dengue, chikungunya, and HFMD followed different pandemic era trajectories within a shared surveillance system. The January and March models estimated the level reductions. The mean annual incidence in 2023–2025 exceeded the pre-pandemic baseline for dengue and HFMD but remained lower for chikungunya. These comparisons support multi-disease surveillance but cannot establish transmission ecology as the cause of the differences. Natural epidemic dynamics, particularly for chikungunya, and unmeasured time-varying factors limit causal interpretation.

## Funding

This research did not receive any specific grant from funding agencies in the public, commercial, or not-for-profit sectors.

## Ethical approval

This study used de-identified aggregated surveillance data and was determined to be exempt from ethical review by the institutional review board of Bamrasnaradura Infectious Diseases Institute (Project number S002 h/69_Exempt; determination dated February 27, 2026). The requirement for informed consent was waived.

## Generative artificial intelligence use statement

The authors used Paperpal (AI) for language editing and ChatGPT (OpenAI) to assist with manuscript organization, language refinement, revision drafting, consistency checks, and code-assisted verification of existing analyses. No synthetic observations were generated. The authors are responsible for reviewing and validating the text, interpretations, and computational checks, and the final content of the manuscript.

## CRediT authorship contribution statement

**Wannarat Pongpirul:** Writing – review & editing, Writing – original draft, Validation, Supervision, Project administration, Investigation, Conceptualization. **Mohamed Mustaf Ahmed:** Writing – review & editing, Writing – original draft, Visualization, Software, Methodology, Investigation, Formal analysis, Data curation. **Krit Pongpirul:** Writing – review & editing, Writing – original draft, Supervision, Resources, Methodology, Investigation, Conceptualization.

## Declaration of competing interest

The authors have no competing interests to declare.

## Appendix Supplementary materials

Image, application 1
