# Supplementary material for: Contrasting pandemic era trajectories of dengue; chikungunya; and hand, foot, and mouth disease in Thailand using national surveillance findings, 2016–2025
Source: IJID Reg. 2026 Oct 7;21:101037. doi: 10.1016/j.ijregi.2026.101037 (PMC13722057; doi:10.1016/j.ijregi.2026.101037)
Supplement: Supplementary file 1 [file mmc1.docx]

**Supplementary Table S1**: STROBE checklist

| Item | Recommendation | Manuscript location or applicability |
| --- | --- | --- |
| 1 | (a) Indicate the study’s design with a commonly used term in the title or the abstract | Abstract, Methods, national ecological interrupted time-series design. |
| 1 | (b) Provide in the abstract an informative and balanced summary of what was done and what was found | Abstract, Objectives, Methods, Results, and Conclusions. |
| 2 | Explain the scientific background and rationale for the investigation being reported | Introduction, paragraphs 1–3. |
| 3 | State specific objectives, including any prespecified hypotheses | Introduction, paragraph 4. |
| 4 | Present key elements of study design early in the paper | Methods, Study design and setting. |
| 5 | Describe the setting, locations, and relevant dates, including periods of recruitment, exposure, follow-up, and data collection | Methods, Study design and setting; Data sources; Outcome definitions. |
| 6 | (a) Give the eligibility criteria, and the sources and methods of selection of participants | Methods, Data sources; Supplementary Methods, Data and descriptive summaries. All available aggregate reporting records were included; no individual recruitment. |
| 7 | Clearly define all outcomes, exposures, predictors, potential confounders, and effect modifiers. Give diagnostic criteria, if applicable | Methods, Outcome definitions and Statistical analysis; limitations identify unmeasured covariates. |
| 8* | For each variable of interest, give sources of data and details of methods of assessment (measurement). Describe comparability of assessment methods if there is more than one group | Methods, Data sources and Outcome definitions. Ministry of Public Health surveillance, Department of Provincial Administration populations, and Royal Thai Survey Department boundary data. |
| 9 | Describe any efforts to address potential sources of bias | Methods, Sensitivity analyses; Discussion, limitations. Date sensitivity and seasonal adjustment do not correct under-ascertainment or time-varying confounding. |
| 10 | Explain how the study size was arrived at | Methods, Data sources; Supplementary Methods, Data and descriptive summaries. Entire available series; no sampled cohort. |
| 11 | Explain how quantitative variables were handled in the analyses. If applicable, describe which groupings were chosen and why | Methods, Outcome definitions and Statistical analysis; Supplementary Methods. |
| 12 | (a) Describe all statistical methods, including those used to control for confounding | Methods, Statistical analysis; Supplementary Methods, Segmented time-series model. Time, seasonality, and population offset included; relevant time-varying covariates unmeasured. |
| 12 | (b) Describe any methods used to examine subgroups and interactions | Disease-specific models and broad-category descriptive comparisons; no formal interaction analysis reported. |
| 12 | (c) Explain how missing data were addressed | Methods, Data sources; Supplementary Methods. No missing province-week records; no imputation. |
| 12 | (d) If applicable, describe analytical methods taking account of sampling strategy | Not applicable. all available surveillance records were used, without a sampling stage. |
| 12 | (e) Describe any sensitivity analyses | Methods, Sensitivity analyses; Results, Sensitivity to alternative interruption dates; Supplementary Table S5. |
| 13* | (a) Report numbers of individuals at each stage of study, eg numbers potentially eligible, examined for eligibility, confirmed eligible, included in the study, completing follow-up, and analysed | Results, Surveillance records and disease groupings. 40,579 province-week records; 77 provinces; 527 reporting weeks; disease totals reported. |
| 13* | (b) Give reasons for non-participation at each stage | Not applicable to individual participation. Reporting-grid completeness does not establish complete ascertainment of infections. |
| 13* | (c) Consider use of a flow diagram | Not applicable. aggregate surveillance time series without individual recruitment stages. |
| 14* | (a) Give characteristics of study participants (eg demographic, clinical, social) and information on exposures and potential confounders | Results, Surveillance records and disease groupings and Geographic distribution; Supplementary Tables S2–S4. Individual demographic and clinical characteristics were not available. |
| 14* | (b) Indicate number of participants with missing data for each variable of interest | Methods, Data sources. No missing province-week records. Individual-level variables were not available. |
| 15* | Report numbers of outcome events or summary measures | Results; Table 1; Supplementary Tables S2–S5. |
| 16 | (a) Give unadjusted estimates and, if applicable, confounder-adjusted estimates and their precision (eg, 95% confidence interval). Make clear which confounders were adjusted for and why they were included | Results, Interrupted time-series analyses; Table 1. Adjustment specification in Methods and Supplementary Methods; unmeasured confounders acknowledged in Discussion. |
| 16 | (b) Report category boundaries when continuous variables were categorized | Methods, Outcome definitions, 2016–2019, 2020–2022, 2023–2025 and date thresholds. |
| 16 | (c) If relevant, consider translating estimates of relative risk into absolute risk for a meaningful time period | Results, National temporal trajectories; Supplementary Table S3. Absolute incidence rates and percentage changes reported. |
| 17 | Report other analyses done, eg analyses of subgroups and interactions, and sensitivity analyses | Results, Sensitivity to alternative interruption dates and Cross-disease temporal associations; Supplementary Table S5 and Figure 5A–B. |
| 18 | Summarise key results with reference to study objectives | Discussion, paragraph 1. |
| 19 | Discuss limitations of the study, taking into account sources of potential bias or imprecision. Discuss both direction and magnitude of any potential bias | Discussion, paragraphs 3 and 7. Timing, epidemic dynamics, unmeasured factors, ascertainment, and serial correlation; direction/magnitude cannot generally be quantified. |
| 20 | Give a cautious overall interpretation of results considering objectives, limitations, multiplicity of analyses, results from similar studies, and other relevant evidence | Discussion, paragraphs 1–7; Conclusion. Causal attribution is explicitly limited. |
| 21 | Discuss the generalisability (external validity) of the study results | Discussion, paragraph 8, implications for Thailand and Southeast Asia; national surveillance setting described in Methods. |
| 22 | Give the source of funding and the role of the funders for the present study and, if applicable, for the original study on which the present article is based | Declarations, Funding source, no specific grant received. |

Note: Information on the STROBE Initiative is available at [www.strobe-statement.org](http://www.strobe-statement.org).

# Supplementary methods

# Data and descriptive summaries

All available province-week records in the analysis workbook were included, including 40,579 records from 77 provinces and 527 reporting weeks during 2016–2025. This was an analysis of the available national surveillance series, not a sampled cohort. No missing province-week records were identified and no imputation was performed. Completeness of the reporting grid does not imply complete ascertainment of infections.

National weekly cases were summed across provinces and divided by the corresponding sum of year-specific provincial registered populations. Annual rates used annual case totals and one population denominator per province-year. Phase-specific national rates and province rankings were based on mean annual rates. Weekly trajectories were smoothed using an 8-week centered moving mean (minimum one observation). Seasonal profiles summarize mean weekly incidence by calendar month, as defined in the caption to Figure 2.

**Segmented time-series model**

For each disease, national weekly counts were fitted with a Poisson generalized linear model using a log link and Pearson-scaled variance (quasi-Poisson inference). The linear predictor included a continuous observation index t, a post-breakpoint indicator, the interaction of that indicator with time since the first included post-breakpoint observation, two sine/cosine harmonics with a 52.1775-week period, and log national population as an offset.

The immediate level IRR is exp(βpost). The reported annualized slope change is 100 × [exp(52βinteraction) − 1]. The absolute post-breakpoint trend would depend on βtime + βinteraction and is not the reported slope-change quantity. Wald confidence intervals use the coefficient estimate ±1.96 standard errors before transformation. Pearson scaling accounts for overdispersion but does not explicitly model residual serial correlation.

The primary threshold was 1 January 2020. Sensitivity thresholds were 22 March 2020 and 12 October 2020. The post indicator used the recorded week-start date ≥ threshold; consequently, the first included observations were 1 January (reporting week 1), 22 March 2020 (reporting week 13), and 18 October (reporting week 43). Earlier labels of weeks 12 and 42 did not match the implemented date thresholds. Supplementary Table S5 reports the original date-based estimates. These thresholds are analytical definitions, not exact policy or epidemic-wave onset dates.

**Seasonal references and cross-disease comparisons**

Seasonal reference models included an intercept and the same two Fourier harmonics, without a time trend or interruption terms. They were fitted to pre-2020 observations for dengue and HFMD and to pre-2018 observations for chikungunya. Predicted counts used the population offset for each observed week. The low-incidence chikungunya reference avoids extrapolating the epidemic upswing but is not a causal no-pandemic counterfactual.

The deviations displayed in Figure 4 were calculated across observations from 1 January 2020 onward as 100 × (sum of observed cases − sum of seasonal-reference counts) / sum of seasonal-reference counts. For chikungunya, the large deviation is displayed as the observed/reference ratio (approximately 200), not as an attributable fraction. The 2020–2022 shading and the 2020–2025 deviation interval serve different purposes.

For Figure 5A, each national weekly incidence series was transformed as log(1 + incidence), then detrended by subtracting its centered 52-week moving mean (minimum 12 observations). Pearson correlations were calculated at lags from −26 to +26 weeks. Positive lag denotes the first named disease preceding the second. The plotted ±1.96/√n band uses n = 527 and is only a nominal reference; serial dependence and multiple lag comparisons are not incorporated. These exploratory correlations do not establish transmission coupling.

For Figure 5B, dengue and chikungunya rates were summed to form the vector-borne category, while HFMD was displayed separately. Each category’s 8-week centered moving mean was divided by its own pre-2020 mean and multiplied by 100. Differences between these broad categories cannot isolate transmission route from differences in age, pathogens, immunity, or epidemic history.

# Supplementary Table S2: Annual case counts and national population, 2016–2025

| Year | Dengue cases | Chikungunya cases | Hand, foot, and mouth disease cases | National population |
| --- | --- | --- | --- | --- |
| 2016 | 64,174 | 18 | 80,348 | 65,931,550 |
| 2017 | 53,961 | 10 | 70,733 | 66,188,503 |
| 2018 | 87,656 | 3,581 | 70,191 | 66,413,979 |
| 2019 | 129,757 | 11,721 | 67,738 | 66,558,935 |
| 2020 | 72,557 | 11,334 | 33,805 | 66,186,727 |
| 2021 | 10,524 | 661 | 19,007 | 66,171,439 |
| 2022 | 46,655 | 1,309 | 100,140 | 66,090,475 |
| 2023 | 160,841 | 1,486 | 65,673 | 66,052,615 |
| 2024 | 105,986 | 740 | 90,923 | 65,951,210 |
| 2025 | 58,152 | 1,405 | 115,264 | 65,809,011 |

Note: Counts are notified cases. Population denominators are year-specific registered populations from the Department of Provincial Administration.

**Supplementary Figure S1.** Annual dengue cases by severity stratum (A) and each stratum’s share of total dengue notifications (B), Thailand, 2016–2025.

# Supplementary Table S3: Mean annual incidence by study period

| Disease | Pre-pandemic rate  (2016–2019) | Pandemic rate  (2020–2022) | Δ% vs Pre-pandemic | Post-pandemic rate  (2023–2025) | Δ% vs Pre-pandemic |
| --- | --- | --- | --- | --- | --- |
| Dengue | 126.4 | 65.4 | −48.3% | 164.2 | +29.8% |
| Chikungunya | 5.8 | 6.7 | +16.3% | 1.8 | −68.1% |
| Hand, foot, and mouth disease | 109.0 | 77.1 | −29.3% | 137.5 | +26.1% |

Note: Rates are mean annual cases per 100,000 population. Percentage changes use unrounded rates relative to 2016–2019. The post-pandemic label is operational and does not imply that COVID-19 effects ended in 2023.

# Supplementary Table S4: Ten provinces with the highest mean annual incidence by disease

| Rank | Dengue province | Dengue rate | Chikungunya province | Chikungunya rate | Hand, foot, and mouth disease province | Hand, foot, and mouth disease rate |
| --- | --- | --- | --- | --- | --- | --- |
| 1 | Mae Hong Son | 504.1 | Chanthaburi | 54.4 | Chanthaburi | 224.9 |
| 2 | Chiang Rai | 239.1 | Songkhla | 30.7 | Chiang Rai | 189.6 |
| 3 | Rayong | 214.7 | Uthai Thani | 30.2 | Phayao | 182.7 |
| 4 | Phuket | 213.6 | Pattani | 27.2 | Phuket | 170.0 |
| 5 | Songkhla | 209.7 | Ranong | 22.3 | Rayong | 164.3 |
| 6 | Chiang Mai | 194.5 | Phuket | 22.0 | Chiang Mai | 161.7 |
| 7 | Phatthalung | 179.2 | Lamphun | 21.6 | Mae Hong Son | 159.3 |
| 8 | Trat | 175.3 | Rayong | 16.0 | Saraburi | 157.0 |
| 9 | Chonburi | 170.8 | Tak | 14.2 | Nan | 153.9 |
| 10 | Phetchaburi | 170.8 | Loei | 13.9 | Bangkok | 150.9 |

Note: Rates are mean annual cases per 100,000 population during 2016–2025.

# Supplementary Table S5: Sensitivity of interrupted time-series estimates to alternative date thresholds

| Disease | Analysis | Date threshold | Level IRR (95% CI) | Level p | Annualized slope change (95% CI) | Slope p |
| --- | --- | --- | --- | --- | --- | --- |
| Dengue | Primary | 2020-01-01 | 0.39 (0.32, 0.48) | <0.001 | -11.4% (-18.2%, -3.9%) | 0.003 |
|  | March sensitivity | 2020-03-22 | 0.39 (0.32, 0.49) | <0.001 | -8.5% (-15.4%, -0.9%) | 0.028 |
|  | October sensitivity | 2020-10-12 | 0.37 (0.30, 0.47) | <0.001 | +8.2% (+0.5%, +16.5%) | 0.037 |
| Chikungunya | Primary | 2020-01-01 | 0.54 (0.41, 0.71) | <0.001 | -85.9% (-88.6%, -82.6%) | <0.001 |
|  | March sensitivity | 2020-03-22 | 0.47 (0.35, 0.62) | <0.001 | -84.2% (-87.1%, -80.6%) | <0.001 |
|  | October sensitivity | 2020-10-12 | 0.05 (0.04, 0.07) | <0.001 | -63.0% (-67.7%, -57.7%) | <0.001 |
| Hand, foot, and mouth disease | Primary | 2020-01-01 | 0.51 (0.38, 0.69) | <0.001 | +41.9% (+28.2%, +57.1%) | <0.001 |
|  | March sensitivity | 2020-03-22 | 0.55 (0.41, 0.75) | <0.001 | +44.1% (+30.4%, +59.2%) | <0.001 |
|  | October sensitivity | 2020-10-12 | 1.30 (0.97, 1.75) | 0.077 | +52.0% (+38.9%, +66.4%) | <0.001 |

Note: IRR, incidence rate ratio; CI, confidence interval. Primary threshold, 1 January 2020. March threshold, 22 March 2020. October threshold, 12 October 2020. First included reporting observations were weeks 1, 13, and 43, respectively (1 January, 22 March, and 18 October). Slope changes are relative to the pre-interruption trend. No threshold identifies a causal intervention effect.
